# A Novel NAC Transcription Factor From *Eucalyptus*, EgNAC141, Positively Regulates Lignin Biosynthesis and Increases Lignin Deposition

**DOI:** 10.3389/fpls.2021.642090

**Published:** 2021-04-08

**Authors:** YiMing Sun, Chunxue Jiang, Ruiqi Jiang, Fengying Wang, Zhenguo Zhang, Jianjun Zeng

**Affiliations:** ^1^School of Life Sciences, Southwest University, Chongqing, China; ^2^Chongqing Three Gorges University College of Public Administration, Chongqing, China; ^3^College of Life Sciences, Shandong Normal University (SDNU), Jinan, China; ^4^School of Life Sciences, Jinggangshan University, Ji’an, China

**Keywords:** *Eucalyptus*, NAC transcription factor, lignin biosynthesis, wood formation, secondary cell wall

## Abstract

Wood formation is a complicated process under the control of a large set of transcription factors. NAC transcription factors are considered “master switches” in this process. However, few NAC members have been cloned and characterized in *Eucalyptus*, which is one of the most economically important woody plants. Here, we reported an NAC transcription factor from *Eucalyptus grandis*, EgNAC141, which has no Arabidopsis orthologs associated with xylogenesis-related processes. *EgNAC141* was predominantly expressed in lignin-rich tissues, such as the stem and xylem. Overexpression of *EgNAC141* in Arabidopsis resulted in stronger lignification, larger xylem, and higher lignin content. The expression of lignin biosynthetic genes in transgenic plants was significantly higher compared with wild-type plants. The transient expression of *EgNAC141* activated the expression of Arabidopsis lignin biosynthetic genes in a dual-luciferase assay. Overall, these results showed that EgNAC141 is a positive regulator of lignin biosynthesis and may help us understand the regulatory mechanism of wood formation.

## Introduction

Woody plants have a rich vascular structure and provide valuable renewable resources for pulp, paper, and bioenergy production (Zhong et al., 2019). In angiosperms, two principle sclerenchyma cell types, xylem vessels and xylary fibers, comprise the secondary xylem, which facilitate the transport of water and nutrients and provide mechanical strength, respectively (Plomion et al., 2001). Various factors including wood density and chemical composition, fiber and vessel length, and cell wall thickness affect pulp yield, digestibility, and quality; however, the relative importance of each of these factors varies among species (Ona et al., 2001; Ramírez et al., 2009). During xylem formation, the cells of secondary xylem differentiated from vascular cambium elongate and deposit a lignified secondary cell wall (SCW). The SCW is primarily composed of lignin, cellulose, and hemicellulose. Various genes involved in the biosynthesis of lignin and cellulose have been identified (Meents et al., 2018), and the transcriptional network underlying SCW biosynthesis has been well studied, especially in the model plant Arabidopsis. Kubo et al. (2005) showed that the plant-specific NAM, ATAF1,2, and CUC2 (NAC) transcription factors (TFs) VASCULAR-RELATED NAC-DOMAIN 6 and 7 (VND6 and VND7) are the master regulators of proto- and metaxylem vessel cell differentiation. Subsequent studies have shown that certain NAC domain TFs are master switches controlling SCW formation, including VND1–VND7, NST1–NST3, and SND1 (Zhong et al., 2006; Mitsuda et al., 2007; Yamaguchi et al., 2008), that directly or indirectly regulate the expression of numerous MYB genes. Thus, MYB TFs are the second-layer master regulators of SCW formation, such as AtMYB58/63/85, which are lignin-specific TFs, and AtMYB26/32/41/44/46/61/83/103, which participate in SCW biosynthesis (Zhong and Ye, 2009, 2014; Camargo et al., 2019).

Over the past decade, studies of the function of NAC TFs in regulating SCW formation have shown that NAC TFs are first-layer master switches in both herbaceous plants, such as Arabidopsis (Kubo et al., 2005; Zhong et al., 2006; Mitsuda et al., 2007; Yamaguchi et al., 2008), and in woody plants, such as poplar (Zhong and Ye, 2010; Ohtani et al., 2011; Zhong et al., 2011; Yang et al., 2019). *Eucalyptus* trees are economically important, fast-growing hardwoods that are primarily used for the pulp and paper industries and cover ca. 20 million hectares worldwide (Forest Genetic Resources, 2000, Food and Agriculture Organization of the United Nations). The fast growth, high yield, and ease of cultivation of *Eucalyptus* make this genus an important feedstock in the forestry sector as well as a producer of second-generation biofuel (Salazar et al., 2016). Aside from providing mechanical support and being used as feedstock, lignin also affects biofuel conversion. Consequently, studying SCW formation in *Eucalyptus*, especially the biosynthesis of lignin, has become a major focus of forestry science research. Studies of the regulation of SCW synthesis by MYB TFs have also been conducted in *Eucalyptus*. EgMYB1 inhibits lignin biosynthesis (Legay et al., 2010), whereas EgMYB2 positively regulates lignin biosynthesis (Goicoechea et al., 2005). Recently, Ployet et al. (2019) identified a new SCW regulator, EgMYB137, using an integrated network-based approach. NAC TFs play a key role in regulating lignin biosynthesis; however, few studies have examined the regulatory roles of NAC TFs in lignin biosynthesis in *Eucalyptus* (Hussey et al., 2015).

The number of NAC TFs varies widely among plant species: 105 in *Arabidopsis thaliana*, 177 in soybean (*Glycine max*), 115 in maize (*Zea mays*), and 138 in rice (*Oryza sativa*) (Hu et al., 2019). A total of 189 NAC TFs have been documented in *Eucalyptus*, 7 of which (EgNAC24, EgNAC32, EgNAC58, EgNAC59, EgNAC90, EgNAC141, and EgNAC157) are encoded by genes that have no *Arabidopsis* orthologs associated with xylogenesis-related processes, such as VND1–VND7, SND1–SND3, and NST2–NST3. Combined with their increased expression in vascular tissues, Hussey et al. (2015) speculated that EgNAC24, EgNAC32, EgNAC90, EgNAC141, and EgNAC157 might play a role in the regulation of xylogenesis-related processes. In this study, we characterized the phylogenetic relationships between these novel candidate TFs and other NAC TFs and analyzed their relative expression levels in different tissues. We then conducted an in-depth study of EgNAC141, which was specifically expressed in the xylem of *Eucalyptus*.

## Materials and Methods

### Plant Materials and Growth Conditions

The surface-sterilized *Arabidopsis thaliana* seeds were planted on MS medium containing 30 g/L sucrose and 10 g/L agar for 15 days. After germination, the seedlings were transferred to nutrient soil and were cultivated in an illumination incubator (20–23°C, 16/8 h light/dark cycle, 10,000 LUX light, and 75% humidity). Tobacco (*Nicotiana benthamiana*) was planted under the same conditions as Arabidopsis; however, a higher temperature (24–28°C) was used for the dual-luciferase assay in tobacco.

Sterile seedlings of *Eucalyptus grandis* (genotypes Hook) were provided by Dr. Xiaojian Qu (Kunming Institute of Botany, Chinese Academy of Sciences) in June 2016. The seedlings were then transferred to 10-L pots with mixed substrate (volume ratio humus/vermiculite/perlite: 5/3/2) and grown in a greenhouse. The seedlings were cultured for 6 months in the greenhouse at 25°C (light/16 h), 20°C (dark/8 h), and 60% humidity before treatment and RNA extraction.

### Cloning of Full-Length EgNAC141 and Vector Construction

Total RNA was extracted with Trizol regent (Invitrogen) and used as a template to synthesize first-strand cDNA by reverse transcription PCR. The coding sequences of *EgNAC141* were amplified with gene-specific primers based on the sequence of *EgNAC141* in the *E. grandis* genome database (Eucgr.I00583) (Supplementary Table 1) and were inserted into the pMD19-T (TaKaRa) vector. After confirmation by sequencing, the fragment was subcloned into the binary vector pBI121, generating construct pBI121-*EgNAC141*, which was then transformed into *Agrobacterium tumefaciens* strain GV3101 via the freeze–thaw method.

### Bioinformatics Analysis

The Phytozome^1^ databases were used to retrieve sequence information for genes in the evolutionary analysis. To identify the NAC family members of *E. grandis*, v2.0 annotated protein sequences of the *E. grandis* genome were downloaded from Phytozome v12.0^2^. Protein sequences with a Pfam NAM domain (PF02365) were recognized by HMMER program version 3.2. All but the longest splice variants were removed. The multiple sequence alignment was carried out using MAFFT program version 7.455 (Katoh and Standley, 2013) with default settings. The maximum likelihood (ML) trees were inferred from concatenated gene sequences using IQ-TREE v.1.6.12 (Nguyen et al., 2015), and the best-fit substitution model was automatically selected using ModelFinder (Kalyaanamoorthy et al., 2017). Bootstrap support was estimated using the ultrafast bootstrap approximation with 1,000 replicates (Hoang et al., 2018) (-bb 1,000 -m MFP). The accession numbers of NAC family members identified in this study using the *E. grandis* genome V2.0 and in the study of Hussey et al. (2015) in which v1.0 annotations were used, as well as the accession numbers of genes of poplar and Arabidopsis, are listed in Supplementary Table 2.

### Generation of *EgNAC141* Overexpression Lines

The pBI121-*EgNAC141* overexpression construct was transformed into *Arabidopsis* (Col-0) using the *A. tumefaciens*-mediated floral dipping method. Transgenic plants (T1) were screened on MS medium (supplemented with 50 mg/L hygromycin and 400 mg/L cefotaxime). Homozygous transgenic lines were selected for further research.

### RNA Extraction and RT-qPCR

Trizol reagent (Invitrogen) was used for RNA extraction from *Arabidopsis* stems and *Eucalyptus* tissues. Genomic DNA was removed by DNase I (Takara) treatment. First-strand cDNA was synthesized using a PrimeScript^TM^ RT MasterMix kit (Takara). Real-time quantitative PCR (RT-qPCR) was performed using Novostar-SYBR Supermix (Novoprotein, Shanghai, China) with the Thermal Cycler Dice Real Time System TP950 (TaKaRa, Japan). The *Actin* gene of Arabidopsis and *Eucalyptus* was used as an internal control. All primer sequences for RT-qPCR analysis are listed in Supplementary Table 1.

### Histochemical Assay

The internode stems were collected from the 30-day-old wild and transgenic Arabidopsis lines for histochemical assays. The internode cross-sections were stained with 0.05% (w/v) toluidine blue reagent (TBO) for lignin characterization as described previously (Li et al., 2018). The micrographs were taken under an Olympus microscope (SZX10, Japan). To quantitatively analyze the changes in xylem area of *EgNAC141*-overexpressing transgenic Arabidopsis, the 4th and 5th internodes of inflorescence stems of 30-day-old Arabidopsis were sliced, and paraffin sections (12 mm) were made with an ultra-thin semiautomatic microtome (KD-3368AM, Kedee, Zhejiang, China) per the manufacturer’s instructions. The paraffin sections were then immediately stained with 0.05% (w/v) TBO for 30 s; temporary slides were observed, and pictures were taken with an optical microscope system. ImageJ was used to calculate the radial width of xylem. There were four biological replicates (25–35 slides per replicate) for both WT (WT) and *EgNAC141*-overexpressing Arabidopsis (L1, L4, and L5).

### Chemical Analysis of Lignin Components

Samples (40 mesh) were screened using a mill and were extracted with benzene/ethanol (2:1, v/v) for 8 h. The resulting meals were used for lignin content determination via the Klason method (Dence, 1992). The components of lignin were measured as previously described (Li et al., 2015).

### Subcellular Localization of EgNAC141

The coding sequence of *EgNAC141* was amplified with gene-specific primers (Supplementary Table 1) and inserted into the binary vector pBI121-GFP to generate the construct pBI121-*EgNAC141*-GFP, in which *EgNAC141* was fused with a *GFP* gene under the control of the 35S promoter. The resulting construct was infiltrated into the tobacco leaves as described previously (Xu et al., 2014) and cultured in the dark. After 48 h, the GFP signal in the infected tobacco leaves was detected using a ZEISS LSM 900 confocal microscope at a wavelength of 488 nm.

### Self-Activation Assay in Yeast

To study the self-activation of *EgNAC141*, the coding sequence of *EgNAC141* was cloned into the vector pGBKT7, generating plasmid pGBKT7-*EgNAC141*, and was transformed into the yeast strain *Saccharomyces cerevisiae* Gold2 by the PEG-LiAc method (Zaragoza et al., 2004). Positive yeast transformants were screened on SD medium lacking Trp (tryptophan) and were transferred to SD medium lacking Trp, His (histidine), and Ade (adenine) for the transactivation assay. The self-activation of *EgNAC141* was also confirmed by adding X-α-gal into SD medium lacking Trp, His, and Ade.

### Transient Expression Activity Assay

Dual-luciferase (dual-LUC) assays were performed as previously described (Zhang et al., 2015). Briefly, the promoter regions of lignin biosynthesis genes (*CCOAOMT1*, *CCR1*, *CSE*, *COMT*, and *CAD1*) from Arabidopsis were amplified using the primers listed in Supplementary Table 1. Subsequently, these promoters were inserted into pGREEN0800 (kindly provided by Fangyuan Zhang, Southwest University) to generate reporter constructs. The pBI121-*EgNAC141* construct was used as the effector. Each pair of effector and reporter constructs was co-infiltrated into the leaves of *N*. *benthamiana*, and the relative LUC expression assay was performed after 48 h per the instructions of the Dual-Luciferase Kit (Promega).

### Statistical Analysis

Data were analyzed using Student’s *t* tests and one-way ANOVAs. Microsoft Excel 2016 (Microsoft Corporation) and Graphpad Prism 8 (Mestrelab Research) were used to conduct statistical analyses and create graphs.

## Results

### EgNAC24/141/157 Were Located in a Eucalyptus Expansion Subfamily

**FIGURE 1:**
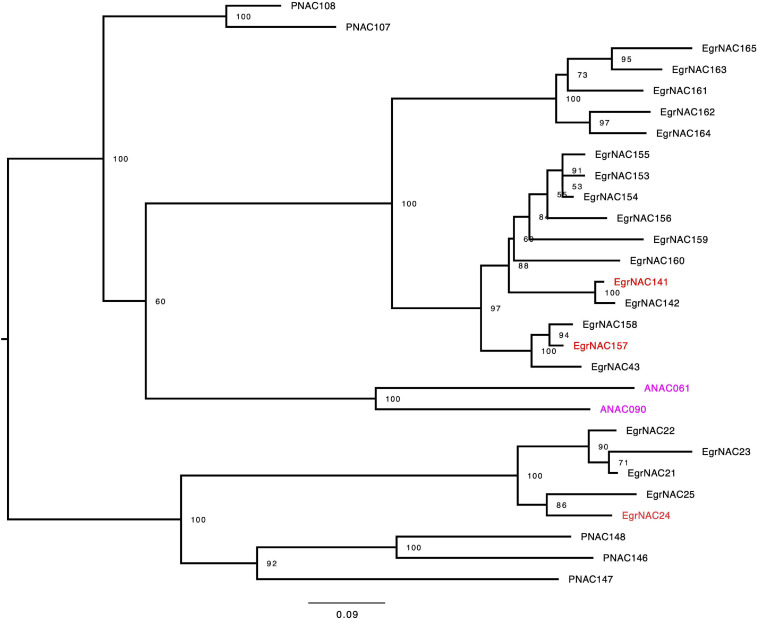
Maximum-likelihood phylogeny of the IVc subfamily of NAC TFs in *Arabidopsis*, Populus, and *Eucalyptus grandis*. Trees were rooted at the midpoint. The values on the nodes were given by ultrafast bootstrap approximation (UFBoot) with 1,000 replicates.

NAC TFs are the master switches of lignin biosynthesis, and lignin is the main component of wood in woody plants. Although some NAC TFs that regulate lignin biosynthesis in angiosperm plants, such as VND1–VND7, SND1–SND3, and NST2–NST3, are conserved, some species-specific or tree-specific NAC TFs might also participate in this process (Hussey et al., 2015). Using both comparative phylogenetics and large-scale expression profiling, Hussey et al. (2015) identified seven NAC TFs (EgrNAC24, EgrNAC32, EgrNAC58, EgrNAC59, EgrNAC90, EgrNAC141, and EgrNAC157) that might play a role in the regulation of xylogenesis-related processes given that they are preferentially expressed in the xylem and have no Arabidopsis orthologs associated with these processes (Hussey et al., 2015). However, in the new version of the *E. grandis* genome annotation (v2.0), the accession numbers of *EgrNAC58* and *EgrNAC59* were not available. Consequently, we could not amplify these two genes through PCR. To obtain experimental data to test their hypothesis, we retrieved NAC family protein sequences and reconstructed a phylogenetic tree using NAC family members of *E. grandis*, poplar, and Arabidopsis. Consistent with the results of Hussey et al. (2015), EgNAC24, EgNAC141, and EgNAC157 were clustered into the IVc subfamily; EgNAC32 and EgNAC90 were located in the Va(1) and IVa subfamily, respectively. As shown in Figure 1, the *Eucalyptus* NAC TF genes exhibited notable expansion in the IVc subfamily. Only two Arabidopsis NAC TFs (ANAC061 and ANAC090) but more than 20 Eucalyptus TFs existed in that subfamily. In addition, few evidence showed that the Arabidopsis orthologs of Eucalyptus NAC TF genes of IVc subfamily, *ANAC061* and *ANAC090*, were involved in regulating SCW biosynthesis so far.

### Tissue Expression Pattern of *EgNAC24/32/90/141/157*

**FIGURE 2:**
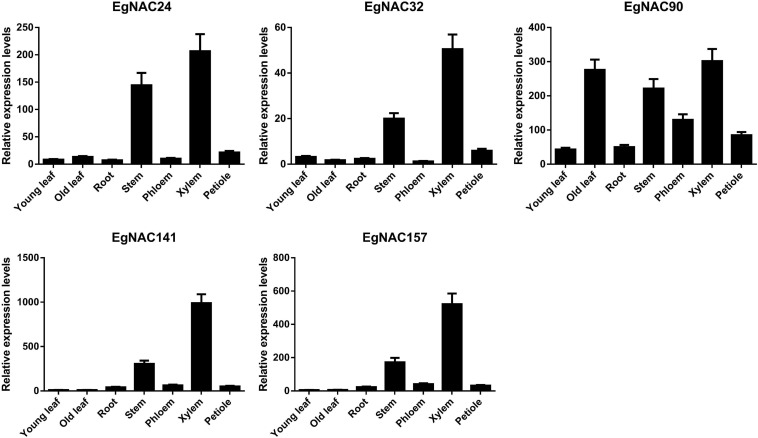
The relative expression levels of *EgNAC24/32/90/141/157* in different tissues of *Eucalyptus grandis*; data represent means ± SD of three replicates.

Tissue- or cell-specific expression is one of the most important characteristics of NAC TFs involved in the regulation of lignin biosynthesis. Real-time quantitative PCR (qRT-PCR) was used to analyze the spatial expression patterns of *EgNAC24/32/90/141/157*. The expression of *EgNAC90* was higher in xylem, old leaf, stem, and phloem (Figure 2), suggesting that EgNAC90 may be involved in multiple biological processes in addition to wood formation. Nevertheless, *EgNAC24/32/141/157* were primarily expressed in xylem; expression levels were markedly higher in the xylem and stem compared with other tissues, including young leaf, old leaf, root, phloem, and petiole tissues, suggesting that EgNAC23/32/141/157 might be involved in the regulation of lignin biosynthesis. In addition, the expression levels of *EgNAC23/32/157* were extremely low in all tissues with the exception of stem and xylem, and the expression level of *EgNAC141* was 1,000 times higher in xylem than in stem. We investigated the function of EgNAC141 further given its xylem-specific expression.

### EgNAC141 Is a Transcriptional Activator That Is Localized in the Nucleus

**FIGURE 3:**
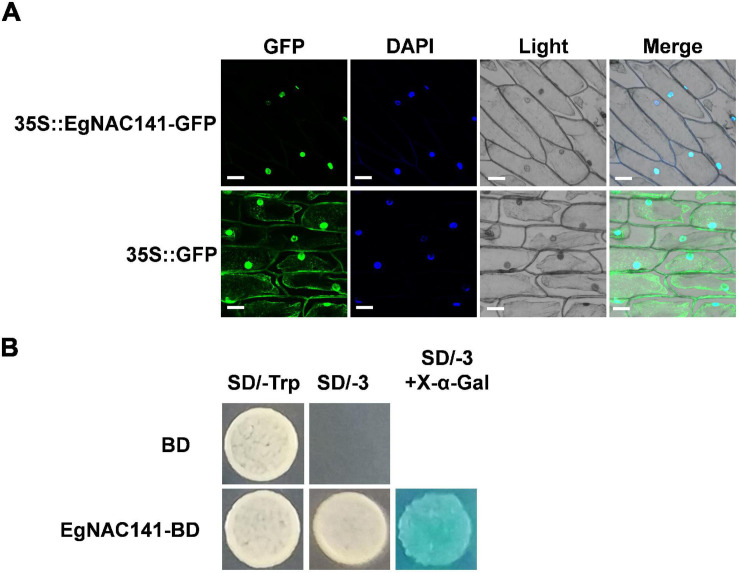
Subcellular localization of EgNAC141 and transcriptional activity assay of EgNAC141. **(A)** Nuclear localization of EgNAC141 detected via transient expression in onion bulb epidermal cells. The nucleus was indicated by DAPI staining. Blank vector 35S-GFP was used as the control. **(B)** Transactivation capability of EgNAC141 tested in yeast cells. The yeast cells expressing GAL4BD-EgNAC141 fusion protein were transferred to a selective medium lacking adenine, histidine, and tryptophan (SD/-AHT) and turned blue after the X-α-gal overlay.

Determining the subcellular localization of EgNAC141 in plant cells is important; given that it is a TF, it is likely localized in the nucleus. We conducted transient expression assays of *EgNAC141* fused to GFP protein in onion leaf using gene gun bombardment. The expression of *EgNAC141*-GFP resulted in a robust green fluorescence colocalized with the blue fluorescence of DAPI staining (Figure 3A). EgNAC141 was localized in the nucleus, consistent with EgNAC141 being a TF. To determine whether EgNAC141 was a transcriptional activator, we fused it with the GAL4-DNA binding domain for transactivation analysis in yeast. Yeast with *EgNAC141* was able to grow on a medium lacking tryptophan, leucine, and histidine and turned blue after X-α-Gal application (Figure 3B), indicating that EgNAC141 is a transcriptional activator.

### Overexpression of *EgNAC141* in Arabidopsis-Induced Lignin Biosynthesis

To further investigate the function of EgNAC141 in plants, an *EgNAC141* overexpression construct was generated and transformed into Arabidopsis by the floral dipping method. After antibiotic screening, 10 independent lines were obtained. PCR of genomic DNA was carried out to confirm the positive transgenic lines. All 10 lines except line 9 were positive transgenic plants (Supplementary Figure 1A). Three homozygous transgenic plants (lines 1, 4, and 5) with higher relative expression were selected by qRT-PCR and used for further study (Supplementary Figure 1B).

**FIGURE 4:**
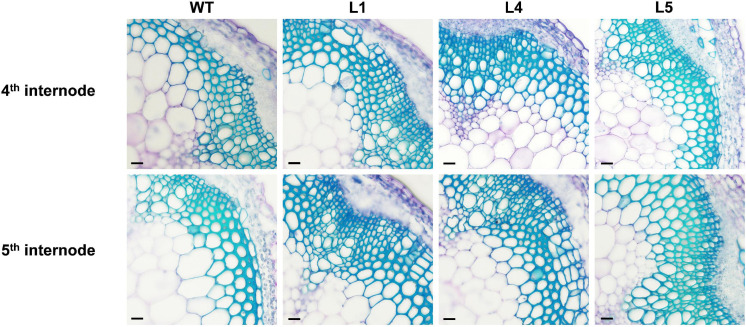
Microscopic analyses of stems from the control and *EgNAC141*-overexpressing Arabidopsis plants. General view of the stem vascular tissues stained by TBO in the 4th and 5th internode of 2-month-old inflorescence stem transverse sections: WT and *EgNAC141*-overexpressing plants (L1, L4, and L5). Each independent line has four replicates, and the micrographs are from biological replicate 1 (R1). The other biological replicates are shown in Supplementary Figure 3. Bars: 500 μm.

There was no significant difference in the phenotypes between the WT and *EgNAC141*-overexpressing transgenic Arabidopsis during the 30-day growth period (Supplementary Figure 2 and Supplementary Table 3). Microscopic observations of hand-sliced sections between sections 4 and 5 of the inflorescence stem stained with TBO showed that the number of xylem cells in *EgNAC141* overexpression lines was significantly increased, resulting in thicker xylem (Figure 4 and Supplementary Figure 8). The lignified area of the transgenic plants increased by 41.32–55.21% in the 4th section and 37.62–67.23% in the 5th section compared with the WT (Table 1). Consistent with histological staining, significantly increased lignin content was observed in *EgNAC141* overexpression lines. All three lines exhibited significant (between 1.2- and 1.5-fold) increases in acid-insoluble lignin content. By contrast, only line 1 had a significantly increased (ca. 1.3-fold) acid-soluble lignin content compared with the WT (Table 2). Finally, the total lignin content that was calculated by adding the acid-soluble and acid-insoluble lignin together significantly increased by approximately 1.2- to 1.4-fold. Overall, these results indicated that *EgNAC141* overexpression positively affected the biosynthesis of lignin, primarily through the increase in acid-insoluble lignin.

**TABLE 1:** Radial width of xylem in inflorescence stems of WT and *EgNAC141*-overexpressing transgenic Arabidopsis plants (*P < 0.05; **P < 0.01).

**Samples**	**Radial width of xylem (μm)**		
		**4th internode**	**5th internode**
WT		317.45 ± 9.36	412.36 ± 11.42
	L1	492.12 ± 30.32**	689.33 ± 45.26**
*EgNAC141*	L4	468.77 ± 24.53*	611.28 ± 39.19**
	L5	447.45 ± 21.38*	567.48 ± 35.46*

**TABLE 2:** Lignin content of WT and *EgNAC141*-overexpressing transgenic plants in the inflorescence stems of Arabidopsis (μg/mg dry weight).

	**WT**	**EgNAC141**		
		**Line 1**	**Line 4**	**Line 5**
Acid-soluble	48.33 ± 3.52	63.72 ± 5.79*	59.01 ± 5.71*	55.42 ± 4.87
Acid-insoluble	78.39 ± 8.06	127.72 ± 11.55**	114.27 ± 11.02**	104.90 ± 9.57*
Total lignin	126.72 ± 11.58	194.44 ± 17.34**	173.28 ± 16.73**	160.32 ± 14.44*

The results are given as means ± SD of four independent replicates (*P < 0.05; **P < 0.01).

**FIGURE 5:**
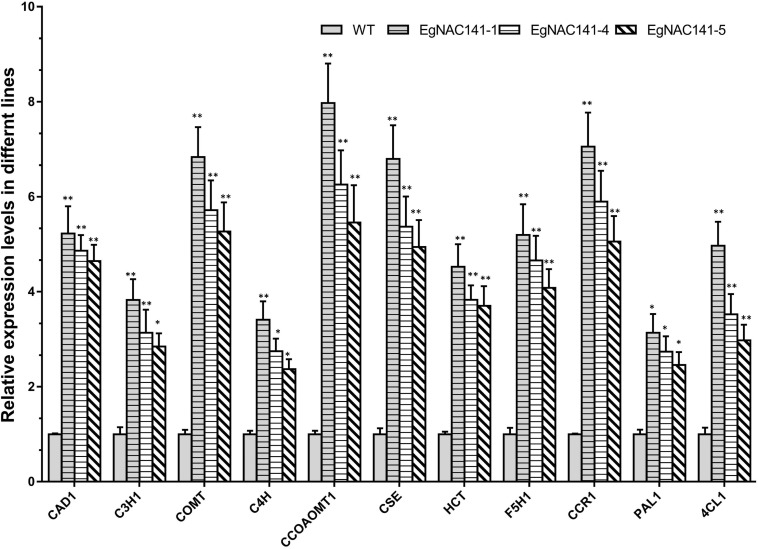
The relative expression levels of Arabidopsis lignin biosynthesis genes in WT and *EgNAC141*-overexpressing Arabidopsis plants. Data are means ± SD of three replicates. Significant differences between means were determined using Student’s *t* tests (*P < 0.05; **P < 0.01).

To assess the role of EgNAC141 in regulating lignin biosynthesis at the transcriptional level, the relative expression levels of 11 genes involved in lignin biosynthesis were quantified by qRT-PCR. Significant increases in the transcript abundances of the genes in all three *EgNAC141* overexpression lines were observed, especially *CCOAMT1*, *COMT*, *CSE*, *CCR1*, and *CAD1* (Figure 5). The observed increase in the expression levels of lignin biosynthesis genes was consistent with the increase in lignin content, suggesting that EgNAC141 transactivates the expression of the lignin biosynthesis pathway in Arabidopsis.

### EgNAC141 Transactivates the Expression of Lignin Biosynthetic Genes

**FIGURE 6:**
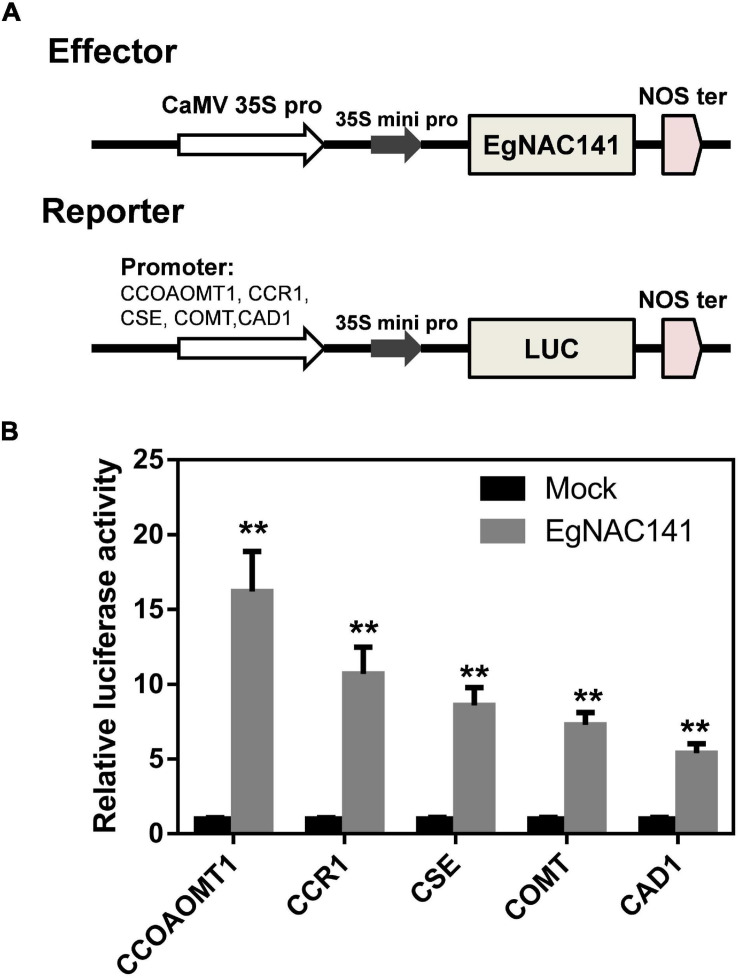
Dual-luciferase assays in leaves of *Nicotiana benthamiana* to study the ability of EgNAC141 to transactivate lignin biosynthesis genes. **(A)** Diagrams of effector and reporter constructs. **(B)** Results of EgNAC141 dual-luciferase assays using the promoters of lignin biosynthesis genes. Data are means (±SE), *n* = 3. Significant differences between means were determined using Student’s *t* tests (**P < 0.01).

We tested the ability of EgNAC141 to directly activate the expression of these genes using a dual-LUC assay. Specifically, the promoters of *CCOAMT1*, *COMT*, *CSE*, *CCR1*, and *CAD1* (the most up-regulated genes) were cloned and inserted into pGreen0800 to drive the expression of luciferase, which generated reporter constructs; the *EgNAC141*-overexpression construct was used as the effector (Figure 6A). The LUC/REN ratio of tobacco leaves expressing *EgNAC141* was dramatically increased compared with the control in which the construct of *YFP* under 35S promoter was used as the effector (Figure 6B). The LUC/REN ratio increased 16-fold for pCCOAOMT1::LUC, 10-fold for pCCR1::LUC, 8-fold for pCSE::LUC, 7-fold for pCOMT::LUC, and 5-fold for pCAD1::LUC when *EgNAC141* was expressed. This indicates that the effect of EgNAC141 on lignin synthesis is achieved by directly activating the expression of downstream genes.

## Discussion

SCW formation is a complex process that requires the coordinated expression of a series of biosynthetic genes (Wang and Dixon, 2012) and thus a complicated transcriptional regulatory network that can activate or inactivate the expression of enzyme genes (Camargo et al., 2019). Over the past decades, a transcriptional regulatory model has been established by primarily using Arabidopsis, in which the NAC and MYB TF families play leading and secondary roles, respectively (Zhong and Ye, 2014). However, the TFs involved in the formation of wood, which is mainly composed of SCW, are poorly known. Indeed, the transcriptional regulatory mechanism of SCW in wood is more complex. The xylem-associated NAC family in *Eucalyptus* and poplar has expanded at the genome level through duplication (Mitsuda et al., 2007; Hu et al., 2010; Hussey et al., 2015). Specifically, seven NAC TFs in *Eucalyptus* without Arabidopsis homologs might be involved in xylogenesis-related processes based on their increased expression in vascular tissue and phylogenetic analysis (Hussey et al., 2015). Consistent with Hussey et al. (2015), the five novel candidates were clearly separated from other NAC TFs. EgNAC24/141/157 were nested within a single clade, suggesting that they are more similar to each other than to EgNAC32 and EgNAC90 (Figure 1). We also performed qRT-PCR analysis to investigate the relative expression levels of candidate NAC TFs. Similar to the results of Hussey et al. (2015), which were based on RNA-seq data, expression was highest in lignin-rich tissues, with the exception of *EgNAC90*. Transcripts of *EgNAC24/32/141/157* were most abundant in xylem and stem compared with the other tissues that were tested; *EgNAC90* had similar expression levels in old leaf, stem, and xylem. The consistency in the expression patterns of *EgNAC24/32/141/157* between our study and Hussey et al. (2015) confirm that these are *Eucalyptus* NAC TFs that merit further study. The expression of *EgNAC141* was 1,000 times higher in xylem and stem compared with other tissues (Figure 2). This was the main reason why *EgNAC141* was selected for further study.

Furthermore, our data indicated that EgNAC141 is a transcriptional activator that is localized in the nucleus. When *EgNAC141* was overexpressed in Arabidopsis, the lignin content and lignified area of the transgenic plant increased. This demonstrated that EgNAC141 played a role in xylem formation. To verify the morphologies and anatomical observation of ectopic expression of *EgNAC141*, we inspected the relative expression of lignin biosynthetic genes in transgenic Arabidopsis by qRT-PCR. The expression levels of 11 genes belonging to the lignin biosynthetic pathway were up-regulated compared with the WT, suggesting that EgNAC141 could transactivate the expression of these genes. The transactivation effect of EgNAC141 on Arabidopsis lignin biosynthetic genes was also confirmed by the dual-LUC assay. Our data have shown that EgNAC141 is a functional NAC TF that positively regulates lignin biosynthesis via transactivation of lignin biosynthetic genes. Most studies have used ectopic expression in Arabidopsis to study *Eucalyptus* TFs using orthologs in other plants given that transgenic protocols for *Eucalyptus* are lacking. For example, Legay et al. (2010) reported that overexpression of *EgMYB1* in Arabidopsis altered vascular development and reduced SCW thickening; Soler et al. (2017) showed that EgMYB1 interacted with EgH1.3, a linker histone variant, which limited the lignin deposited in xylem cell walls in Arabidopsis. Navarrete-Campos et al. found that overexpression of Arabidopsis *CBF* homologous genes in *Eucalyptus*, *EgCBFs*, and Arabidopsis improved the freezing tolerance of transgenic Arabidopsis (Navarrete-Campos et al., 2017). Hence, our results indicated that EgNAC141 positively regulates lignin biosynthesis in plants; however, whether EgNAC141 has a similar positive regulatory function in *Eucalyptus* and Arabidopsis remains unclear. Although there is little information on the differences between the regulatory networks underlying xylem development in herbaceous and woody plants, the little evidence that has been obtained to date has suggested that these networks can vary. For example, the overexpression of *AtSND2* in Arabidopsis increased xylem fiber wall thickness (Zhong et al., 2008), whereas the overexpression of an *SND2* ortholog in poplar, *PopNAC154*, resulted in an increase in the proportion of bark versus xylem in poplar tree and did not affect SCW thickness (Grant et al., 2010).

One issue requiring clarification is why the overexpression of some *E. grandis* genes lacking Arabidopsis orthologs associated with xylogenesis-related processes led to induced lignin accumulation in Arabidopsis. Legay et al. (2010) reported that the overexpression of *EgMYB1*, an R2R3 MYB from *Eucalyptus*, produces similar phenotypes in Arabidopsis and poplar, including fewer lignified fibers, reduced SCW thickening, and lower Klason lignin content. However, the protein sequence identity between EgMYB1 and AtMYB4, the closest ortholog in Arabidopsis, was 58%, which is similar to the identity between EgNAC141 and AtMYB90 (54%). EgMYB1 is a close ortholog of AtMYB4; however, AtMYB4 has never been reported to regulate SCW synthesis but is known to regulate the accumulation of sinapate esters through its direct target cinnamate 4-hydroxylase. Recently, Agarwal et al. (2020) showed that AtMYB4 also regulates cadmium tolerance by directly binding to the promoters of phytochelatin synthase 1 (*PCS1*) and metallothionein 1C (*MT1C*) genes (Agarwal et al., 2020). Few studies have examined the function of AtNAC90, the closest ortholog of EgNAC141 in Arabidopsis, and this work has revealed that AtNAC90 negatively regulates leaf senescence by suppressing the SA and ROS responses (Kim et al., 2018). The evolution of NAC90 and EgNAC141 might reflect the retention of an ancestral function of regulating lignin biosynthesis; however, AtNAC90 has not yet been observed to be involved in this process. Alternatively, the different functions might reflect a rapid expansion of this family/clade that has caused paralogs in Myrtaceae to acquire new functions via neofunctionalization.

In sum, this study shows that EgNAC141 acts as a transcriptional activator to promote lignin biosynthesis by activating the expression of biosynthesis genes. Overexpression of *EgNAC141* increased the number of layers of xylem vessels and the xylem region in transgenic Arabidopsis stems. Our results supported the hypothesis of Hussey et al. (2015) and contributed new insight into the similarities and differences in the molecular mechanisms underlying lignin biosynthesis between Arabidopsis and *Eucalyptus*.

## Data Availability Statement

The datasets presented in this study can be found in online repositories. The names of the repository/repositories and accession number(s) can be found in the article/Supplementary Material.

## Author Contributions

JZ and ZZ designed this work. YS, CJ, and RJ performed the experiments, analyzed the data, and drafted the manuscript. FW and ZZ analyzed the data and drafted the manuscript. JZ revised the manuscript. All authors approved the final revision of the manuscript for publication.

## Conflict of Interest

The authors declare that the research was conducted in the absence of any commercial or financial relationships that could be construed as a potential conflict of interest.

## Footnotes

1 https://phytozome.jgi.doe.gov/pz/portal.html

2 http://www.phytozome.net
