# Supplementary material for: A Novel NAC Transcription Factor From Eucalyptus, EgNAC141, Positively Regulates Lignin Biosynthesis and Increases Lignin Deposition
Source: Front Plant Sci. 2021 Apr 8;12:642090. doi: 10.3389/fpls.2021.642090 (PMC8061705; doi:10.3389/fpls.2021.642090)
Supplement: Supplementary file 3 [file Table_3.DOCX]

Supplementary Table. Plant height of wild type and EgNAC141-overexpression transgenic Arabidopsis (cm).

|  | WT |  | EgNAC141 |  |
| --- | --- | --- | --- | --- |
|  |  | Line1 | Line4 | Line5 |
| Plant height | 26.18±3.58 | 24.72±3.34 | 25.49±3.73 | 24.29±4.07 |

Plant materials were collected from the stems of 30-day-old plants. The results are given as means ±SD of 4 independent replicates. Significant difference was analyzed by Student’s t-test.
