# Supplementary material for: A Novel NAC Transcription Factor From Eucalyptus, EgNAC141, Positively Regulates Lignin Biosynthesis and Increases Lignin Deposition
Source: Front Plant Sci. 2021 Apr 8;12:642090. doi: 10.3389/fpls.2021.642090 (PMC8061705; doi:10.3389/fpls.2021.642090)
Supplement: Supplementary file 4 [file Data_Sheet_1.docx]

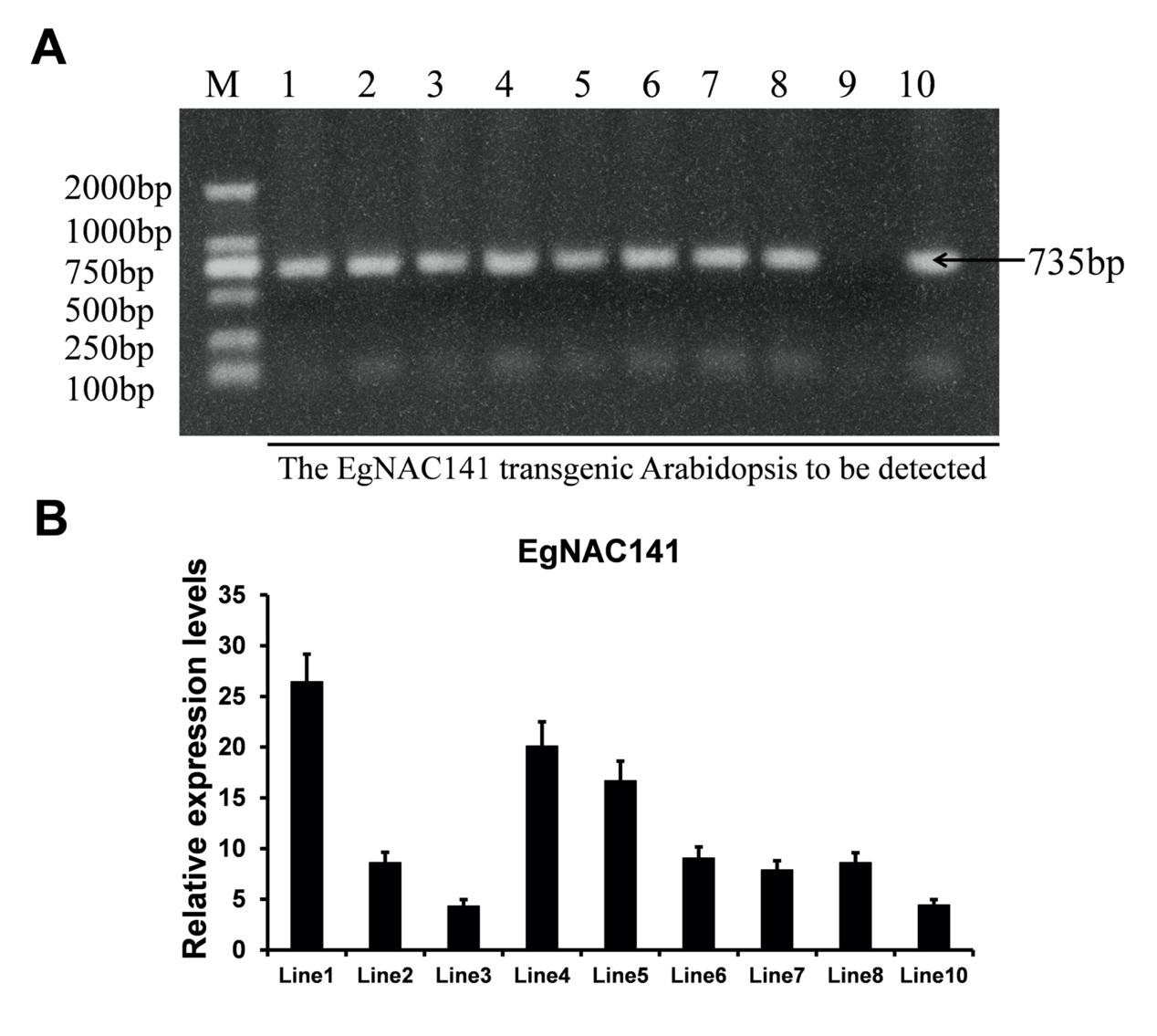


Supplementary Figure 1. A, The genomic-PCR detection of EgNAC141 in transgenic Arabidopsis. B, The qRT-PCR analysis of EgNAC141 in transgenic Arabidopsis.


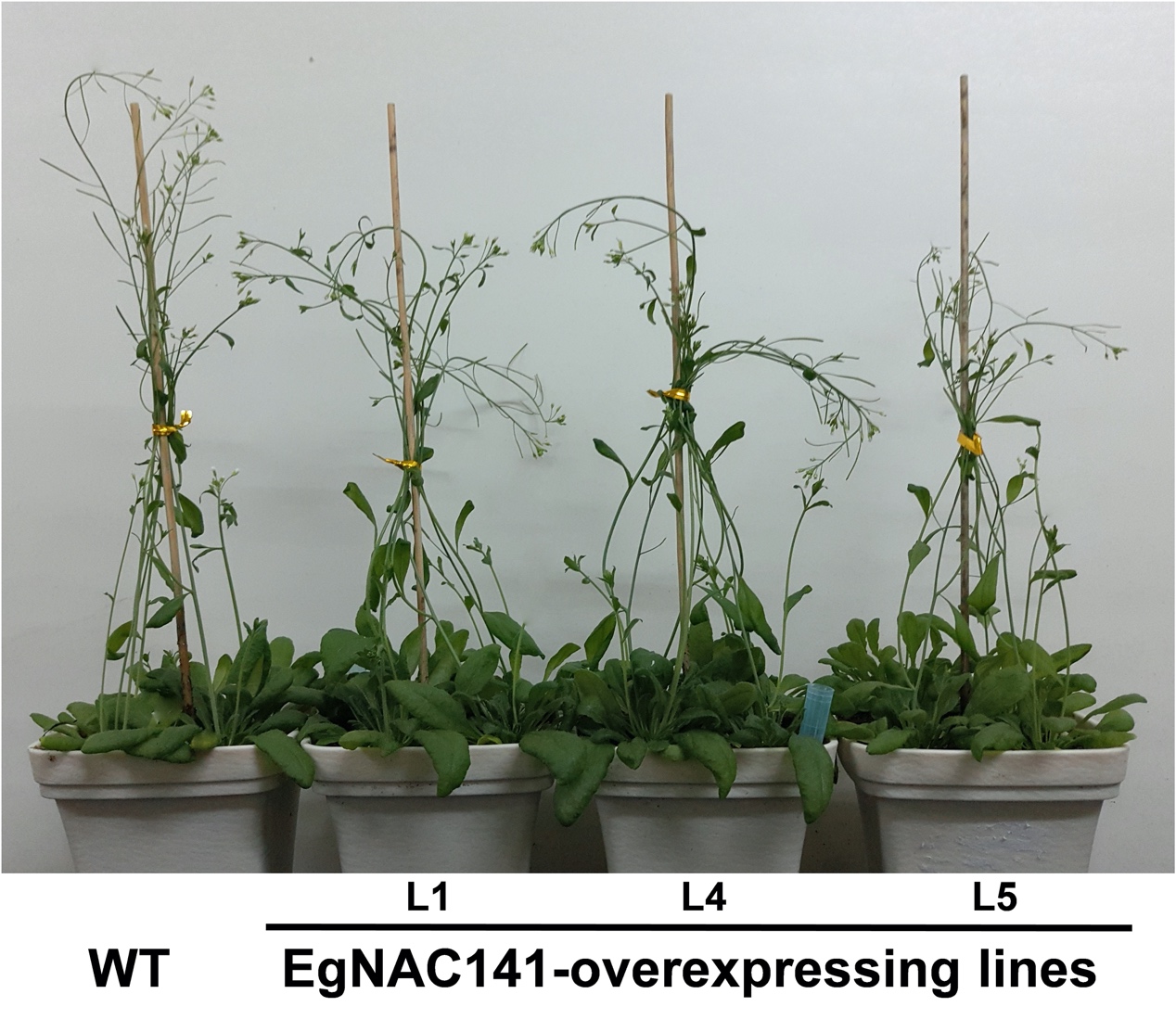


Supplementary Figure 2. The phenotype of 30-day old EgNAC141-Overexpression Arabidopsis.


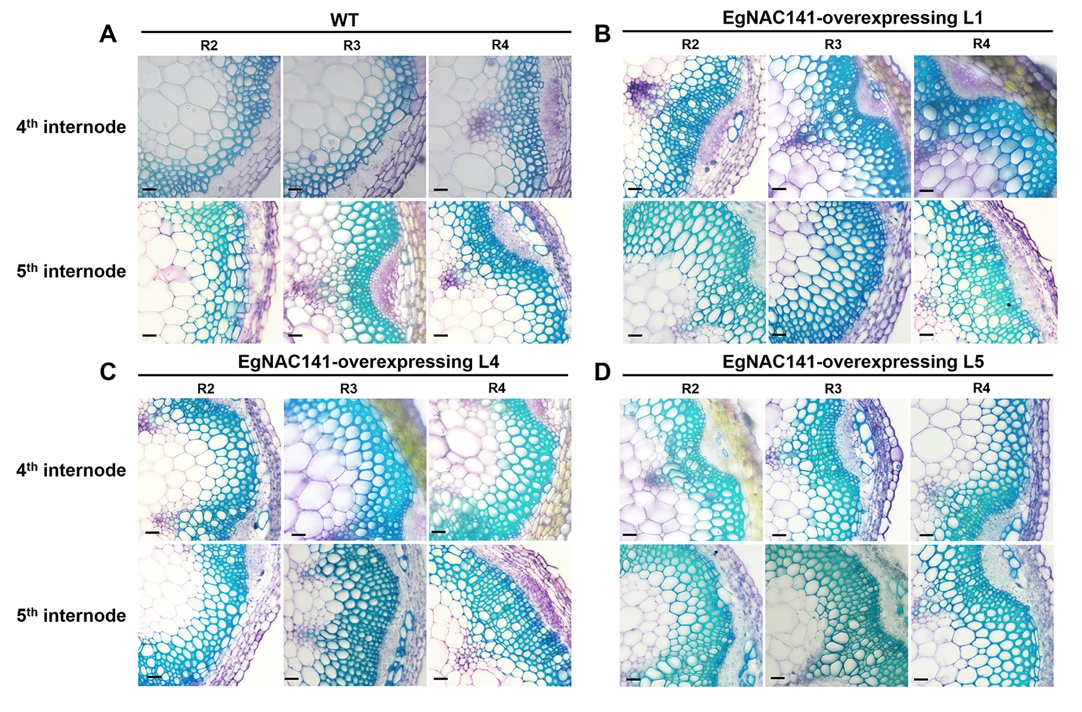


Supplementary Figure 3. ﻿Microscopic analyses of stems from the control and *EgNAC141*-overexpressing Arabidopsis plants. ﻿General view of the stem vascular tissues stained by TBO in the 4^th^ and 5^th^ internode of 2-month-old inflorescence stem transverse sections: WT and *EgNAC141*-overexpressing plants (L1, L4, and L5). R2-R4 represent biologic repeats.
